# Personalized and Self-Management: Systematic Search and Evaluation Quality Factors and User Preference of Drug Reference Apps in Taiwan

**DOI:** 10.3390/jpm11080790

**Published:** 2021-08-12

**Authors:** Yu-Chun Chen, Wei-Wei Liao, Mei-Chin Su, Yen-Hsi Lin

**Affiliations:** 1 Department of Family Medicine, Taipei Veterans General Hospital, Taipei 112, Taiwan; 2 School of Medicine, National Yang Ming Chiao Tung University, Taipei 112, Taiwan; sonia1999082727@yahoo.com; 3 Institute of Hospital and Health Care Administration, National Yang-Ming Chiao Tung University, Taipei 112, Taiwan; sandy102032052@gmail.com; 4 Department of Nursing, Taipei Veterans General Hospital, Taipei 112, Taiwan; mcsu@vghtpe.gov.tw

**Keywords:** drug reference app, keyword growing and systematic search, Mobile App Rating Scale

## Abstract

Background: Drug reference apps promote self-management and improve the efficiency and quality of work for physicians, nurses, pharmacists, and patients. This study aimed to describe a systematic and stepwise process to identify drug reference apps in Taiwan, assess the quality of these apps, and analyze the influential factors for user ratings. Methods: A two-step algorithm (KESS) consisting of keyword growing and systematic search was proposed. Seven independent reviewers were trained to evaluate these apps using Mobile App Rating Scale (MARS). A logistic regression model was fitted and average marginal effects (AME) were calculated to identify the effects of factors for higher user ratings. Results: A total of 23 drug reference apps in Taiwan were identified and analyzed. Generally, these drug reference apps were evaluated as acceptable quality with an average MARS score of 3.23. Higher user engagement, more functionality, better aesthetics, and more information associated with higher user ratings. Navigation is the most influential factor on higher user ratings (AME: 13.15%) followed by performance (AME: 11.03%), visual appeal (AME: 10.87%), credibility (AME: 10.67%), and quantity of information (AME: 10.42%). Conclusions: User experience and information clearly affect user ratings of drug reference apps. Five key factors should be considered when designing drug reference apps.

## 1. Introduction

Drug reference apps have already become a must-have tool for health providers and patients through the whole medication therapeutic cycle [1,2,3,4,5]. While mobile health apps are mostly for reference purposes, drug reference apps are the apps that help users gain a better understanding of medications by providing up-to-date drug information including drug names, trade names, category, classification, mechanism of action, pharmacokinetics, availability, indications and dosages, contraindications, interactions, side effects, serious reactions, and precautions and considerations are ranked within the top 3 most-used apps among medical professionals [1]. There are many studies show that drug reference apps have played a wide variety of roles as clinical decision supporting tools at the point of care for medical professionals [6], as referencing and learning tools for medical students and junior doctors [7,8], and also as standardized dictionaries for inter-professional collaboration [9]. Moreover, drug reference apps could be an interface with up-to-date information written for the layperson that effectively promotes patient education for nurses [10,11] and pharmacists [4,12].

Drug reference apps could empower patients’ self-management, improve medication safety, and optimize therapeutic effect [13,14]. Many people found mobile apps easy to use, that they help in managing medication, and increase treatment adherence in patients by helping them to better understand their medicine [15]. However, it has been proved that medication errors can be aggravated when there are several people involved in the patient’s care by providing accessible, comprehensive, reliable, and understandable medication information to those largely lacking from their health providers [2,16]. The more knowledge of the medication being taken, the more significant the reduction in errors can be [17]. Recent studies also showed that drug reference apps are helpful for medically complex patients such as the elderly [17], multi-comorbidities [18,19], home care patients [20], and also helpful for increasing safety in care transitions [21].

A drug reference app with localized information is vital for improving the therapeutic medication cycle [22,23]. Although there had already dozens of key players, such as Lexicomp, Epocrates, Micromedex, Up-to-date, Drugs.com, etc., frequently mentioned by medical professionals [1,5], these drug reference databases mainly cover medication that is available in the U.S. and Europe and there are very few drug reference apps that provide localized language outside of the U.S. and Europe [23]. For example, a recent report showed that few (15%) hospitals in Taiwan, a country with high medical service availability, provide drug information to their patients, resulting in the limitation for patients’ understanding of their medication [22].

Although there is a need for users and app developers to evaluate the quality of drug reference apps objectively, there is a paucity of information on how they differ, how many and which features they have, their overall quality, and whether they are attractive to users. Previous reviews identified medication adherence-related apps and described their relevant features [2,24,25,26,27,28]. However, these reviews included only the apps in English, without the local ones in Taiwan. This study aimed to describe a systematic and stepwise process to identify drug reference apps with local drug information in Taiwan, assess the quality of these apps by using a reliable quality assessment tool, and analyze influential factors for higher user ratings.

## 2. Materials and Methods

### 2.1. Overview

Our work offers three major contributions. First, we developed a searching algorithm combining keyword growing and systematic searches to extensively retrieve drug reference apps from the Google Play Store and the Apple App Store. We systematically searched, screened, and identified smartphone apps aimed at drug reference in Taiwan. Second, we evaluated and assessed the qualities of these drug reference apps based on the Mobile App Rating Scale (MARS) [29,30]. Last, we gathered users’ feedback and analyzed influential factors for higher user ratings.

### 2.2. Searching Algorithm (KESS) for Drug Reference App: Keyword Growing and Systematic Search

**Figure 1:**
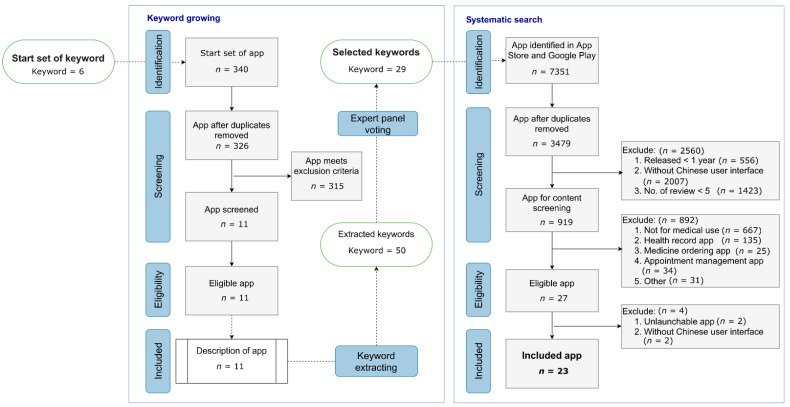
Flow diagram of KESS (Keyword growing and Systematic Search) algorithm and drug reference apps included in this study. (*n* = 23, Taiwan, 2021).

We developed an algorithm (KESS) consisting of two-steps including (1) keyword growing: gathering keywords iteratively and accumulatively from experts and apps’ descriptions; (2) systematic search: using gathered keywords to extensively retrieve apps relevant to drug reference, and then perform a critical review on these app for further analysis (Figure 1).

#### 2.2.1. Keyword Growing

The number and quality of searching result of apps depend heavily on keywords while limited keywords may lead to a biased analysis [31]. To obtain a comprehensive, objective, and unbiased keyword list for drug refence app, we employed a method based on the snowballing technique that was widely used in literature search tasks in conducting systematic reviews [32,33]. The snowballing method may increase the yield of search results by 2.5–43% [34]. The detailed process is available in Appendix A.

#### 2.2.2. Systematic Search

All apps retrieved from the search were screened by two independent reviewers (YCC and WWL) for eligibility using prespecified inclusion and exclusion criteria (Figure 1). The PRISMA (Preferred Reporting Items for Systematic Reviews and Meta-Analysis) guidelines were adopted through the review process that aimed to identify relevant apps that were accessible to most of the general public relevant to drug reference purposes [35].

### 2.3. Quality Appraisal of Apps

All apps that fit the inclusion criteria were evaluated for quality using MARS, which allows reviewers to provide a standardized and objective appraisal across four sections: (1) engagement, including individual items for entertainment, interest, customization, interactivity, and whether the app was engaging for the target users; (2) functionality, including performance, ease of use, navigation, and gestural design; (3) aesthetics, including layout, graphics, and visual appeal; and (4) information quality, including accuracy of app description; whether the app had specific, measurable, and achievable goals; quality of information; quantity of information; visual information; credibility; and whether the app was evidence-based [30,36]. Each item of MARS was evaluated and assigned a 5-point Likert scale rating (1: inadequate, 2: poor, 3: acceptable, 4: good, and 5: excellent) by a panel of reviewers.

In total, 7 independent reviewers (2 health professionals, 2 registered nurses, and 3 laypersons familiar with health apps) were invited and trained to use the MARS instruments through a web-based training program created by the MARS developers [30]. Each reviewer was required to independently test each app on iOS and Android devices for at least 10 min to accomplish a predefined task. All MARS items were translated into Chinese for internal consistency, and we calculated the means of every item and the MARS from all reviewers.

### 2.4. User Ratings

We used the user rating of every app as a proxy of user preference since it is well known that ratings and reviews greatly influence people deciding whether they should or should not download or use an app [37]. Both the App Store and Google Play Store encourage users to express their sentiment towards an app by rating it from 1 to 5 stars, 5 being the best rating, and writing a review in which users put into words what they like or dislike about it, or even ask for new features. Nowadays, apps are rated heavily and the credibility of online ratings was considered as an indicator of users’ preferences for apps. We extracted public user ratings of every app from the App Store or Google Play Store and dichotomized apps into “apps with lower user star ratings (user rating < 4 stars)” and “apps with higher user star ratings (user rating ≥ 4 stars)”.

### 2.5. Data and Statistical Analysis

Further data were collected and analyzed using MedCalc Statistical Software version 19.2.6 (Ostend, Belgium, 2020) and IBM SPSS Statistics for Windows, version 26.0. (Armonk, NY, USA, 2020). We compared the quality factors of MARS for apps with higher user star ratings to those with lower user star ratings using chi-square and *t*-test. Moreover, a logistic regression model with backward elimination was used to assess the influential quality factor for higher user star ratings. *p*-values < 0.05 were considered statistically significant. To compare the impact of each item of MARS on higher user ratings, average marginal effects (AME) of each item were computed by the average of the effect of an item for every observation of its observed value, and 95% confidence intervals were calculated.

## 3. Results

### 3.1. Drug Reference Apps in Taiwan

Twenty-three drug reference apps in Taiwan were included after keyword growing and systematic search (KESS algorithm) (Figure 1). The number of keywords increased from 6 as the *start set* to 29 as the selected set using the keyword growing method. The results of the App Store and Google Play Store searches using the *selected set* of keywords for drug reference app yielded 7351 apps (3479 distinct apps) that were subjected to critical review through PRISMA guidelines, resulted in 23 apps (refer to Figure 1 for a flowchart of the exclusion process). A comprehensive list of drug reference apps in Taiwan is shown in Supplement Table S1, with their respective properties.

Table 1 shows the characteristics of drug reference apps in Taiwan. While all drug reference apps were freeware, two-third (65.2%) of these apps were available on the Google Play Store. Most (69.6%) drug reference apps were classified in the medical category, followed by health and fitness (17.4%), and tools/utilities (13.0%). More than half of these apps were developed by hospitals (56.5%), followed by private groups (34.8%), and universities (8.7%).

Less than half of drug reference apps in Taiwan were rated higher than 4 stars, and the average user star rating of these apps was 3.69 out of 5 stars. Only two apps had more than 50,000 downloads (Table 1). Moreover, the average MARS score of these drug reference apps was 3.23 (ranging between 2.28–4.12). AIGIA pharmacist had the highest MARS score at 4.12 (Supplement Table S1). The majority (78.3%) of apps obtained a MARS score higher than 3, indicating that they provide an acceptable user experience and at least some technical functional value for the users (Table 1).

### 3.2. Apps with Higher User Ratings vs. Lower Ratings

Drug reference apps with higher user star ratings had higher quality scores than those with lower user star ratings (Table 2). Either developer type or primary category had no association with user star ratings. The average MARS score of apps with higher user star ratings was significantly higher than that of apps with lower user star ratings (3.38 vs. 3.05, *p* < 0.001). Apps with higher user star ratings had higher scores for each individual section of the MARS than those with lower user star ratings, which indicated apps with higher user star ratings had higher engagement (2.70 vs. 2.50, *p* = 0.005), functionality (3.85 vs. 3.49, *p* = 0.003), aesthetics (3.39 vs. 2.98, *p* < 0.001), and information (3.55 vs. 3.25, *p* = 0.005).

**Figure 2:**
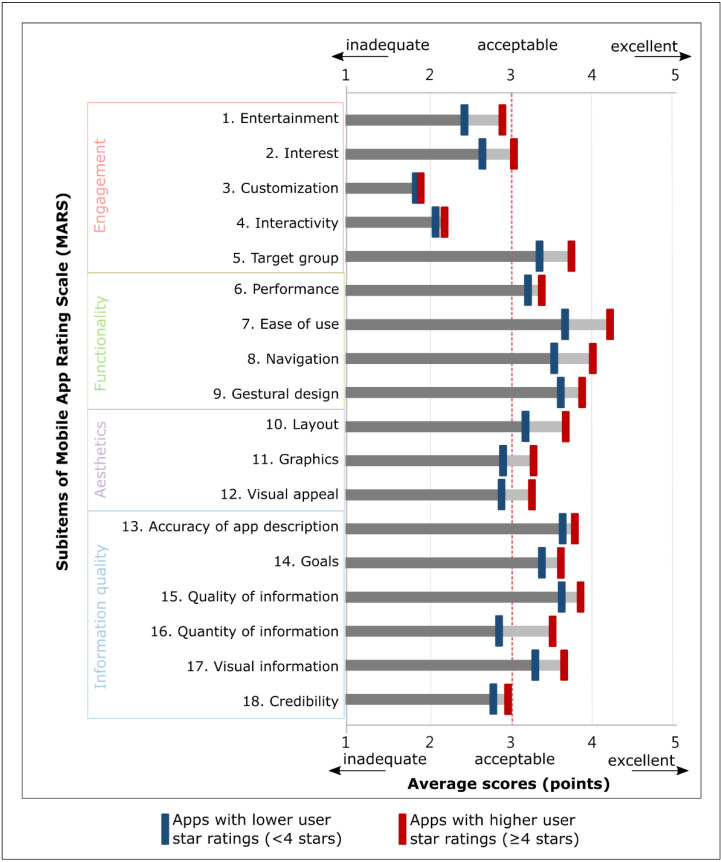
Average scores of items in Mobile App Rating Scale (MARS) of drug reference apps (*n* = 23. Taiwan, 2021). Each horizontal bar denotes an item of MARS. The blue vertical point represents each item’s average score of drug reference apps with lower user star ratings (user ratings < 4 stars), whereas the red vertical point represents the average score of apps with higher user star ratings (user ratings ≥ 4 stars). The light gray bar between two vertical points represents the difference of average score between apps with higher user ratings and those with lower user ratings.

Figure 2 illustrates the average scores of each item in MARS of drug reference apps as well as the difference in average score between apps with higher user ratings and those with lower user ratings. Generally, the items in the engagement section, scored below 3 points (item numbered 1–4), were lower than items in other sections (Figure 2). The finding suggests that these apps are lacking entertainment (item numbered 1 and item numbered 2), interactivity (item number 3), and insufficient customization (item number 4) to appeal to users (Figure 2).

Five out of 18 items of MARS were independently associated with higher user star ratings with different levels of impacts (Table 3). After fitting a binary logistic regression model using backward elimination, navigation and performance in the functionality section, visual appeal in the aesthetics, and credibility and quantity of information positively associate with higher user star ratings (Table 3). Among these five influential items, navigation had the most significant impact on higher user ratings with its AME of 13.15%, which means every increment of navigation score would increase the probability of a higher user star app by 13.15% (the maximal value is 100%), i.e., the chance of users voting high would increase 13.15%. Following navigation, the remaining four items had different levels of impact on higher user rating (AME of performance, 11.03%; visual appeal, 10.87%; credibility, 10.67%; and quantity of information, 10.42%) (Table 3).

## 4. Discussion

Drug reference apps could provide comprehensive localized drug information that play various distinct and vital roles to significantly improve the efficiency and quality of work for physicians, nurses, pharmacists, and patients. The current study aimed to describe a systematic and stepwise process to identify drug reference apps with local drug information in Taiwan, assess the quality of the apps by a reliable quality assessment tool, and analyze the influential factors for higher user ratings.

The two-step algorithm (KESS) consisting of keyword growing and systematic search was proposed. A total of 23 drug reference apps in Taiwan were identified and analyzed. Generally, these drug reference apps were evaluated as acceptable quality apps with the average MARS score of 3.23. Higher engagement, more functionality, better aesthetics, and more information were associated with higher user ratings. Furthermore, the regression model showed that among the four elements, there are five predominantly influential factors, navigation, performance, visual appeal, credibility, and quantity of information.

The proposed KESS algorithm could be a valuable and unbiased framework for systematic searches for apps. Although collecting and identifying suitable apps is the most crucial part and directedly affects the quality of the current study, a standard method or guideline to obtain apps sharing relevant topics is still under research [38]. The first step of the KESS algorithm is very similar to “keyword growing” in the process of conducting systematic reviews [31]. We used a natural language processing toolkit to analyze the descriptions of apps to generate new keywords. This method effectively increased the number of keywords for drug reference apps from 6 to 29. Because of the rapidly changing nature of app market dynamics, this approach would reduce the time effort to capture new keywords (e.g., new slang words), and keep the search strategy updated and unbiased [34,38]. The second step of the KESS algorithm is conducted based on PRISMA guidelines, the intent is to ensure the research is transparent and completed. Since the search results of the Google Play and App Store may vary by users, time, and geo-locations, we found the checklist of PRISMA guidelines very useful to verify that each of the app-searching processes are completely reported and reproducible. Therefore, the KESS algorithm may be an automating research tool which focuses on simplifying and streamlining the process of searching and collecting the apps [39].

User experiences are the determinant of user ratings. Our result clearly showed that navigation, performance, visual appeal, credibility, and quantity of information were the main factors leading to higher user ratings. The finding is compatible with a previous systematic review on barriers and facilitators of information-seeking behaviors [40]. Time is a pain point for information seeking. As an information-seeking tool, the primary purpose of the drug reference apps is to provide a proper amount of drug information within a reasonable response time. Besides hardware performance and software engineering, an optimized user interface and organized, accurate, and concise information would better support such cognitively demanding tasks [41,42]. Our finding showed that users’ ratings would be impacted if the time pain point was alleviated.

There is room for improvement in engagement for drug reference apps in Taiwan. Our results showed that the lack of entertainment, interactivity, and customization in app designs would affect the appeal to users. To keep users using the app, app developers may need to identify potential users, understand their preferences, and ensure that the apps cater to the requirements of each market segment [43]. Gamification is a growing trend that can improve patients’ knowledge, skills, and satisfaction effectively, further encouraging better medication management and adherence of patients [44]. The drug reference app designers, whose primary target audience is the general public, may consider incorporating some gaming/gamification factors in their future designs.

Quality and quantity of information are also vital for higher user ratings. Readability and quality were considered the critical elements of drug information [45]. Human-factor designs, such as the best format, layout, or amount of information, vary among different user groups, thus the drug reference apps should be carefully designed to fit the distinct needs of the different user groups. For example, clinicians have the requirement of easy, and timely access to drug information, such as dosage and usage. Patients had a high demand for updated and tailored information, such as effects and adverse effects for improving their knowledge and understanding of the treatment and health outcomes [46,47]. Moreover, cognitive ergonomics is necessary. For visually impaired users [48], the creative use of pictures can help them communicate better [49]. Furthermore, for the elderly [50], pictograms are the easiest way for them to better understand. In comparison to traditional paper material, drug reference apps should aim to be a personalized support tool, which can provide pertinent information in the right way at the right time [2].

A mismatch between target market and market segment should exist in the drug reference app market in Taiwan. Although iOS devices have about half the market share in Taiwan (market share of iOS vs. Android; 53% vs. 46%; https://gs.statcounter.com/os-market-share/mobile/taiwan, accessed on 6 August 2021), only one-third of drug reference apps support these devices. There might be an opportunity to fill the gap for drug reference apps in iOS devices without excluding commercial entities. However, such findings could be a sign of a moribund market for drug refence apps in Taiwan. We found that all drug reference apps are freemium, probably as a result of the public’s free mentality (the tendency to intuitively expect that all digital services are and should be available at no cost) toward health services in Taiwan [51]. Our findings provide information to create rigid demands that may spur on the drug reference app market in Taiwan.

## 5. Limitation

This current study had some limitations. First, the quality of drug information may not be thoroughly evaluated. Although there are dozens of tools developed explicitly for rating medication leaflets and quality of health website information, there is no instrument for evaluating drug reference apps yet [45]. To evaluate the quality of drug information, this current study used MARS, a general-purpose evaluation tool for apps, to evaluate the information from the drug reference apps after performing the same pre-defined task. This approach may ensure inter-rater consistency, but some opinions from readability and some quality dimensions might be insufficient. Second, under-representation bias resulting from reviewers might exist. However, the high cost of training and conducting MARS surveys resulted in convenience sampling being the best feasible option in the current study. While we tried to invite reviewers from different backgrounds, the result may not represent the whole population well. Third, bias resulting from the learning effect might exist. Cognitive fatigue might exist for app reviewers when performing a series of tests. This issue should be minimized since the reviewers were asked to evaluate these drug apps in random order. Fourth, there might be an under-reporting bias for user ratings. While we used user ratings as an objective measure gathered from the general public, those who have an extreme experience are more likely to review than those who have a moderate experience and this may distort the review. This issue should be minimal because such under-reporting bias is usually prominent only for extremely poor or extremely good quality products. This should not be the case in the current study since most drug reference apps had average user ratings.

## 6. Conclusions

The proposed KESS algorithm could be a valuable and unbiased framework for systematic searches for apps. While user ratings are clearly affected by user experience and information, the users may prefer drug reference apps with higher engagement, more functionality, better aesthetics, and more information. Health providers and app designers should pay special attention to the five most influential factors including navigation, performance, visual appeal, credibility, and quantity of information when developing drug refence apps.

## Figures and Tables

**Table 1:** Characteristics of drug reference apps in Taiwan. (*n* = 23, Taiwan, 2021).

Characteristics	*n* = 23	(%)
App store		
Google Play Store	15	(65.2)
Apple App Store	8	(34.8)
Primary category/genre		
Medical	16	(69.6)
Health and fitness	4	(17.4)
Tools/Utilities	3	(13.0)
Developer type		
Hospital	13	(56.5)
Private group	8	(34.8)
University	2	(8.7)
User feedbacks		
User star rating (star)		
Average (SD ^2^)	3.65	(0.95)
≤2	5	(21.7)
3	8	(34.8)
4–5	10	(43.5)
No. of reviews		
<50	14	(60.9)
50–100	6	(26.1)
>100	3	(13.0)
No. of downloads ^1^		
500–5000	5	(33.3)
5000–10,000	2	(13.3)
10,000–50,000	5	(33.3)
>50,000	3	(20.0)
MARS score ^3^		
≥4: good or above	1	(4.3)
3: acceptable	18	(78.3)
≤2: poor	4	(17.4)

^1^ Only apps in Google Play Store were shown here since the numbers of downloads were not publicly provided by App Store. ^2^ SD: Standard Deviation. ^3^ MARS: Mobile App Rating Scale.

**Table 2:** Characteristics and Mobile App Rating Scale (MARS) scores of drug reference app stratified by user star ratings. (*n* = 23, Taiwan, 2021).

	App with Higher User Star Rating (≥4 Stars)		App with Lower User Star Rating (<4 Stars)			
	*n* = 10	(%)	*n* = 13	(%)	*p* Values	Sig. ^1^
Developer type					0.241	
Hospital	5	(50.0)	8	(61.5)		
Private group	3	(30.0)	5	(38.5)		
University	2	(20.0)				
Primary category/genre					0.276	
Medical	6	(60.0)	10	(76.9)		
Health and fitness	2	(20.0)	1	(7.7)		
Tools/Utilities	2	(20.0)	2	(15.4)		
Quality of app						
MARS ^2^ score (average, SD ^3^)	3.38	(0.64)	3.05	(0.64)	<0.001	***
*Engagement*	2.70	(0.60)	2.50	(0.60)	0.005	**
*Functionality*	3.85	(0.78)	3.49	(0.78)	0.003	**
*Aesthetics*	3.39	(0.77)	2.98	(0.83)	<0.001	***
*Information quality*	3.55	(0.82)	3.25	(0.86)	0.005	**

^1^ Significance: ** *p* < 0.01; *** *p* < 0.001; ^2^ MARS: Mobile App Rating Scale; ^3^ SD: standard deviation.

**Table 3:** Adjusted odds ratio (aOR) and average marginal effects (AME) of items of Mobile App Rating Scale (MARS) associated with higher user star rating ordered by AME for higher user star rating ^1^ (*n* = 23, Taiwan, 2021).

	Adjusted Odds Ratio (aOR) for Higher User Ratings			Average Marginal Effects (AME) for Higher User Ratings		
Items of MARS	aOR	(95% CI ^2^)	*p*-Value	AME (%)	(95% CI ^2^)	*p*-Value
Navigation	2.18	(1.23–3.86)	0.008	13.15	(3.32–22.98)	0.009
Performance	2.07	(1.25–3.44)	0.005	11.03	(2.50–19.56)	0.005
Visual appeal	1.83	(1.11–3.04)	0.018	10.87	(1.57–20.18)	0.022
Credibility	1.79	(1.04–3.08)	0.035	10.67	(1.30–20.04)	0.015
Quantity of information	1.77	(1.25–2.52)	0.001	10.42	(4.41–16.43)	0.001

^1^ A logistic regression model with backward elimination was fitted. All 18 items of MARS were included in the initial model and five items listed were included in the final model. For each item of MARS, AMEs were computed by the average of the effect of an item for every observation at its observed value of such item. ^2^ CI: confidence interval.

## Data Availability

The data presented in this study are available in supplementary material.

## Supplementary Materials

The following are available online at https://www.mdpi.com/article/10.3390/jpm11080790/s1, Table S1: Drug reference apps included in this study ranked by MARS score (*n* = 23, Taiwan, 2021).

## Appendix A. Details of Searching Algorithm (KESS) for Drug Reference App

### Appendix A.1. Start Set of Keywords

The left part of Figure 1 demonstrates the whole process of keyword growing. We started our keyword growing from a small seed set of keywords defined by authors (YCC and WWL), called the *start set* of keywords. We searched two main app stores: Google Play Store and Apple App Store with the app store country and region set to Taiwan between 1 and 15 March 2021, using 6 keywords from the start set (Box A1).

Box A1 *Start set* of keywords for drug reference app in Taiwan. Searched app stores: Google Play Store and Apple App Store Search keywords: 藥 (drug), 藥物 (medicine), 藥丸 (drug pill), 吃藥 (taking medicine), 用藥 (medication list), 處方 (prescription) Inclusion criteria: App store country and region set to Taiwan Exclusion criteria: Apps without Chinese description

### Appendix A.2. Keyword Extracting

After deduplication and removal of ineligible apps, we downloaded descriptions of each eligible app for keyword extraction. We used a natural language processing toolkit to perform text mining tasks, including word segmentation and part-of-speech tagging (a.k.a. POS tagging) [52]. We calculated the word frequency of each word tagged as a noun and picked the top 50 words as extracted keywords for expert panel voting.

### Appendix A.3. Expert Panel Voting

To grade the validity of each extracted keyword, we invited 11 experts, including health professionals, caregivers, and patients familiar with health apps to take a vote for best recall (the degree of relevance of keyword and drug reference app). Finally, we picked keywords with a simple majority (i.e., number of votes > 5) as the selected keyword for drug reference apps in Taiwan.

Box A2 Selected keywords for drug reference apps in Taiwan. (Number of keywords = 29). Searched app stores: Google Play Store and Apple App Store Search keywords: 用藥資訊 (drug information),藥名 (drug name), 藥 (drug), 藥品資訊 (medication information), 用藥安全 (drug safety),  藥物成分 (drug ingredient),西藥 (western medicine),副作用 (side effects), 症狀 (symptoms), 查詢藥品 (search for medication), 用藥 (medication list), 藥物 (medicine), 領藥 (refill medication), 藥品 (medication), 處方 (prescription), 藥品外觀 (outlook of drug), 劑量 (drug dose), 交互作用 (interaction), 處方用藥 (prescription drug), 藥單 (prescription), 處方箋 (prescription), 用藥紀錄 (drug history),  用藥知識 (drug literacy), 正確用藥 (proper use of medication), 食藥署 (Food and Drug Agency), 藥廠 (pharmaceutic company), 治療 (treatment), 藥丸 (drug pill), 吃藥 (taking medicine) Inclusion criteria: App store country and region set to Taiwan Exclusion criteria: Released > 1 year Apps without Chinese description Number of user review < 5
