# Supplementary material for: Personalized and Self-Management: Systematic Search and Evaluation Quality Factors and User Preference of Drug Reference Apps in Taiwan
Source: J Pers Med. 2021 Aug 12;11(8):790. doi: 10.3390/jpm11080790 (PMC8400514; doi:10.3390/jpm11080790)
Supplement: Supplementary file 1 [file jpm-11-00790-s001.zip › jpm-1315311-supplementary.pdf]

Supplement Table S1. Drug reference apps included in this study ranked by MARS score (*n* = 23, Taiwan, 2021).

Rank, Title of App	App Profile, Description, and URL			
**1.愛家小藥師（iOS)**	**Release Year**	**Genre**	**User Ratings**	**MARS Score**
**Little pharmacist**	2019	Utilities	4.10	4.12
**Description** [1]	App integrates the current Taiwan Health Insurance drug information and provides you with easy-to-understand interface to quickly get detailed information about your medications. Provides drug name, keywords, ingredients, appearance, color, etc. Multi-language translation: Provides translation into four languages, including English, Indonesian, Vietnamese, and Malay.			
**URL**	https://apps.apple.com/tw/app/%E6%84%9B%E5%AE%B6%E5%B0%8F%E8%97%A5%E5%B8%AB/id1489142715?uo=4			
**2. 愛家小藥師（Android)**	**Release year**	**Genre**	**User ratings**	**MARS score**
**Little pharmacist**	2019	Tools	4.17	3.85
**Description**[1]	App integrates the current Taiwan Health Insurance drug information and provides you with easy-to-understand interface to quickly get detailed information about your medications. Provides drug name, keywords, ingredients, appearance, color, etc. Multi-language translation: Provides translation into four languages, including English, Indonesian, Vietnamese, and Malay.			
**URL**	https://play.google.com/store/apps/details?id=com.aigia.health.pharmacistfree&hl=zh-TW&gl=tw			
**3. 國泰醫院（iOS)**	**Release year**	**Genre**	**User ratings**	**MARS score**
**App of Cathy hospital**	2013	Medical	2.76	3.82
**Description** [1]	Get to know Cathay: To let you know more about Cathay Hospital before you come to the hospital. Mobile Registration: Allows you to check the outpatient schedule and appointment status, such as whether the appointment is full or not. Medication information: Provides information on the scientific name, product name, specifications, color, storage method, and instructions for medication administration.			
**URL**	https://apps.apple.com/tw/app/%E5%9C%8B%E6%B3%B0%E9%86%AB%E9%99%A2/id1460340159			
**4. 臺中榮民總醫院行動掛號（Android)**	**Release year**	**Genre**	**User ratings**	**MARS score**
**App of TVGH**	2012	Medical	4.40	3.75
**Description** [1]	Taichung Veterans General Hospital Mobile Registration is a full-service mobile registration system that allows people to use smartphones and tablet computers to register. The service items are as follows: Medical information, prescription information, traffic guide, doctor's expertise, appointment status, progress of outpatient consultation, and more. The information that can be queried includes: department, doctor, time, consultation room, location, consultation number and estimated time of arrival, etc. It is a comprehensive mobile registration service that allowing people to enjoy easy and convenient medical consultation services anytime and anywhere. The system is available in the U.S. and Canada. This function allows people to easily complete their medical bills by scanning the barcode on the bill or by individual. After completing SMS authentication, you can check your mobile payment history within three months.			
**URL**	https://play.google.com/store/apps/details?id=tw.com.cidt.vghtc&hl=zh-TW&gl=tw			
**5. 嘉義基督教醫院（Android)**	**Release year**	**Genre**	**User ratings**	**MARS score**
**App of Chia-Yi Christian Hospital**	2016	Health and Fitness	4.24	3.66
**Description** [1]	Christian Hospital Mobile Registration App Provides the following functions: booking appointments, real-time progress check and appointment cancellation.			
**URL**	https://play.google.com/store/apps/details?id=com.cychapp&hl=zh-TW&gl=tw			
**6. 臺中榮民總醫院行動掛號（iOS)**	**Release year**	**Genre**	**User ratings**	**MARS score**
**App of TVGH**	2012	Medical	3.44	3.57
**Description** [1]	App provides the following services: Medical Guide, Traffic Guide, Health Education Information, Reservation Service, Mobile registration, and Appointment Inquiry. The app is free to download and use. Use the App to access all the services you need to get the most out of the			

Rank, Title of App	App Profile, Description, and URL			
	app. It's free to use for up to 24 hours. It has been downloaded more than 100,000 times in the U.S. since its release in 2007. It is free for users to use until the end of the month, and there are no plans to stop using it. Mobile payment. 7-1: This function allows people to easily complete their medical bills by scanning the barcode on the bill or by individual. Location guide function: The indoor location guide function can guide you to the place you want to go according to the building, floor or clinic number.			
URL	https://apps.apple.com/tw/app/%E8%87%BA%E4%B8%AD%E6%A6%AE%E6%B0%91%E7%B8%BD%E9%86%AB%E9%99%A2%E8%A1%8C%E5%8B%95%E6%8E%9B%E8%99%9F/id545244802?uo=4			
7. 國泰醫院 (Android)	Release year	Genre	User ratings	MARS score
App of Cathy hospital	2019	Medical	4.33	3.42
Description [1]	New features include personalized login, multiple registration and mobile payment. Search for the doctor you want to see more quickly by providing common symptoms, department classification, doctor's name and quick registration. Parking lot information and navigation around the hospital to enhance the convenience of transportation. This version only supports Android 5.0 or above "Vitals" or above. .			
URL	https://play.google.com/store/apps/details?id=org.cgh.app&hl=zh-TW&gl=tw			
8. KingDS藥鋪子 (Android)	Release year	Genre	User ratings	MARS score
KingDS pharmacy	2016	Medical	4.49	3.42
Description [1]	Open this pharmacy app and you can find out as soon as you open it, with a friendly interface as easy as chatting with friends. Check the drug information and find out the effect, treatment items, side effects, contraindications, appearance and more. Forgot what medications you have checked before? When you open the search history in the upper right corner, you can find the name of the medication you have checked before.			
URL	https://play.google.com/store/apps/details?id=com.kingnet.App.DrugStore&hl=zh-TW&gl=tw			
9, 醫藥大全 – 臺灣藥品資料庫 (iOS)	Release year	Genre	User ratings	MARS score
Encyclopedia of Medicine in Taiwan	2015	Medical	5.00	3.42
Description [1]	300,000 common drugs and drug ingredients are included in the app. Users can search for drugs with the same ingredients.			
URL	https://apps.apple.com/tw/app/%E9%86%AB%E8%97%A5%E5%A4%A7%E5%85%A8-%E8%87%BA%E7%81%A3%E8%97%A5%E5%93%81%E8%B3%87%E6%96%99%E5%BA%AB/id1009159817?uo=4			
10. 成大醫院藥你健康 (Android)	Release year	Genre	User ratings	MARS score
App of National Cheng Kung University Hospital	2016	Medical	3.92	3.39
Description [1]	Cheng Kung University Hospital Medication Health offers various functions such as drug information, prescription medication, health education garden and medication reminder. The services are described as follows. The hospital also has a registration system app, which provides functions like booking appointments and checking the progress of medical appointments. The functions are designed to provide convenient drug information inquiry services and help the public manage their own medication. It also provides a comprehensive management and reminder for multiple people to take care of their own and their families' medication safety. Users are recommended to install			
URL	https://play.google.com/store/apps/details?id=org.zywx.wbpalmstar.widgetone.uex11455183&hl=zh-TW&gl=tw			
11. 亞東醫點通 (Android)	Release year	Genre	User ratings	MARS score
App of Far Eastern Memorial Hospital	2012	Medical	3.01	3.36
Description [1]	You can also complete the procedures of making outpatient appointments, registering as an agent, collecting medications, checking drug information, and inquiring about the elaborate services of A.T. Medical Point. For more information, please refer to the privacy statement on the official website.			
URL	https://play.google.com/store/apps/details?id=com.femh.fareasternapp&hl=zh-TW&gl=tw			
12. 振興醫院電子處方集 (Android)	Release year	Genre	User ratings	MARS score

Rank, Title of App	App Profile, Description, and URL			
App of Cheng-Hsin General Hospital	2013	Medical	4.53	3.35
Description [1]	The electronic prescription set of Chun Hing Hospital will be downloaded from the mobile device. The contents of the drug information refer to the drug formulary approved by the Ministry of Health and Welfare, iMINS and Micromedx.			
URL	https://play.google.com/store/apps/details?id=tw.org.chgh.med&hl=zh-TW&gl=tw			
13. 仁愛醫院（Android)	Release year	Genre	User ratings	MARS score
App of Ren-Ai hospital	2019	Medical	3.94	3.31
Description [1]	App allows you to make appointments, inquire about medication usage, refer to medical conditions, introduce doctors, and provide transportation guidance to hospitals. You can also set up a list of relatives to provide your elders or children at home with convenient and quick assistance in making appointments or inquiring about medical appointments and receiving medication. The app can be used to make online appointments for medications, make inquiries about the progress of medical appointments, and more.			
URL	https://play.google.com/store/apps/details?id=tw.org.jahreg&hl=zh-TW&gl=tw			
14. 中山醫院（Android)	Release year	Genre	User ratings	MARS score
App of Chung Shan Hospital	2014	Medical	2.86	3.12
Description [1]	Zhongshan Hospital is a comprehensive regional hospital founded in 1976. It has attracted 200 top medical experts from China to participate in its work. It provides services in 31 different medical specialties.			
URL	https://play.google.com/store/apps/details?id=com.frihed.hospital.register.CSHSD&hl=zh-TW&gl=tw			
15. 中國醫點通（Android)	Release year	Genre	User ratings	MARS score
App of China Medical University Hospital	2014	Medical	3.35	3.10
Description[1]	App allows you to complete outpatient appointments and receive medication through your smart mobile device. App can also make use of the voice input function of smart mobile devices. App will automatically remind you of your arrival time, medication collection time and examination time through the cell phone setting. Members can only register as patients of the hospital, so that they can enjoy self-health management services at home, including medical calendar, health education, medication, examination and self- health records. We recommend downloading and installing the latest version of the software.			
URL	https://play.google.com/store/apps/details?id=app.cmuh.org.tw&hl=zh-TW&gl=tw			
16. 藥掃描2.0 (Android)	Release year	Genre	User ratings	MARS score
Medication Scanner 2.0	2019	Tools	1.27	3.06
Description [1]	The FDA has launched a drug scanning app that allows people to search for information about drugs in real time. The current six functions include: drug scanning, drug news, certain drug knowledge, drug license inquiry, rumor dispelling Q&A, and locating a pharmacy. People can also search for nearby pharmacies via GPS and Wi-Fi on their smart devices.			
URL	https://play.google.com/store/apps/details?id=com.ares.medscanapp.Standard&hl=zh-TW&gl=tw			
17. 藥掃描2.0 (iOS)	Release year	Genre	User ratings	MARS score
Medication Scanner 2.0	2016	Utilities	3.57	3.03
Description [1]	The FDA has launched a drug scanning app that allows people to search for information about drugs in real time. The current six functions include: drug scanning, drug news, certain drug knowledge, drug license inquiry, rumor dispelling Q&A, and locating a pharmacy. People can also search for nearby pharmacies via GPS and Wi-Fi on their smart devices.			
URL	https://apps.apple.com/tw/app/%E8%97%A5%E6%8E%83%E6%8F%8F2-0/id1147526405?uo=4			
18. KingDS藥鋪子（iOS)	Release year	Genre	User ratings	MARS score
KingDS pharmacy	2016	Medical	3.96	3.00
Description [1]	Open this pharmacy app and you can find out as soon as you open it, with a friendly interface as easy as chatting with friends. Check the drug information and find out the effect, treatment items, side effects, contraindications, appearance and more. Forgot what medications you have checked before? When you open the search history in the upper right corner, you can found out the name of the medication you have check before.			

Rank, Title of App	App Profile, Description, and URL			
URL	https://apps.apple.com/tw/app/kingds%E8%97%A5%E9%8B%AA%E5%AD%90/id1162046459?uo=4			
19. 中國醫點通（iOS）	Release year	Genre	User ratings	MARS score
App of China Medical University Hospital	2014	Medical	2.21	2.73
Description [1]	App allows you to complete outpatient appointments and receive medication through your smart mobile device. The APP can also make use of the voice input function of smart mobile devices. The system will automatically remind you of your appointment time, medication collection time and examination time through the cell phone setting. Members only can sign up for our membership, so that members can enjoy self-health management services at home, including medical calendar, health education, medication, examination, and self- health records. The app will collect your personal information, including name, ID number, date of birth and contact number.			
URL	https://apps.apple.com/tw/app/%E4%B8%AD%E5%9C%8B%E9%86%AB%E9%BB%9E%E9%80%9A/id834761206?uo=4			
20. 義大醫療行動服務系統（Android)	Release year	Genre	User ratings	MARS score
App of E-Da Hospital	2015	Medical	4.00	2.72
Description [1]	The hospital's main functions are as follows: All medical services are available in the palm of your hand, allowing you to make an appointment on your mobile device. The Yih-Dai Mobile Service System APP provides mobile appointment booking and progress checking, and is integrated with the hospital's state-of-the-art information system.			
URL	https://play.google.com/store/apps/details?id=com.mtech.hospital&hl=zh-TW&gl=tw			
21. I-MED健康照護APP (Android)	Release year	Genre	User ratings	MARS score
I-MED health care app	2016	Health and Fitness	4.99	2.57
Description [1]	The most convenient and fast medication reminder app ever! Just scan the QR code on the medication bag and you can set up daily medication reminders for every meal at once. There are also a variety of useful functions, so let I-MED become your little helper of health. The number of partner pharmacies is increasing rapidly. Please remember to ask the pharmacy for a QR code medicine bag. The detailed list can be found in the APP. The QRcode scanning function is enhanced, you can directly scan the hospital prescription.			
URL	https://play.google.com/store/apps/details?id=com.newmedicine.main&hl=zh-TW&gl=tw			
22. 常用藥物查詢網站（Android)	Release year	Genre	User ratings	MARS score
Frequently used medication	2014	Health and Fitness	3.77	2.30
Description [1]	Links to commonly used drug search sites in Hong Kong and Taiwan are integrated for reference. The information contained in this application is for general information only. No representation, representation, warranty or guarantee, express or implied, is given as to its accuracy or appropriateness for use.			
URL	https://play.google.com/store/apps/details?id=com.andromo.dev137874.app362010&hl=zh-TW&gl=tw			
23. 醫字天書（iOS)	Release year	Genre	User ratings	MARS score
Reference book for doctors	2016	Medical	1.71	2.28
Description [1]	Pregnancy medication reminder, a good helper for moms and babies. Nearly 100,000 common medications and more than 10,000 major ingredients. The top 50 most commonly used drugs to master the most common disease medication.			
URL	https://apps.apple.com/tw/app/%E9%86%AB%E5%AD%97%E5%A4%A9%E6%9B%B8/id1073918701?uo=4			

[1] Description of app was machine translated from traditional Chinese to English using DeepL (https://www.deepl.com/translator) and then summarized by QuillBot (https://quillbot.com/summarize) for international readership. Reviewers of this study were asked to rate each app by reading its original context in Chinese.
